# Goitre géant dyspnéisant

**DOI:** 10.11604/pamj.2013.14.82.2499

**Published:** 2013-03-05

**Authors:** Mohammed Ridal, Mohamed Noreddine Alami

**Affiliations:** 1 Service ORL, CHU Hassan II, Faculté de médecine de Fès, Université Sidi Mohammed Ben Abdellah, Maroc

**Keywords:** Goitre dyspnéisant, goitre géant, thyroïdectomie, hypertrophie thyroïdienne bénigne, Goitre multinodulaire, goiter, giant goiter, thyroidectomy, benign thyroid hypertrophy, multinodular goiter

## Image en medicine

Une femme de 72 ans habitant la compagne, suivie pour une hypertension artérielle, ayant comme antécédents des goitres bénins dans la famille. Elle consulte aux urgences pour une aggravation de sa dyspnée même au repos et une dysphagie haute aux solides. L’examen clinique trouve un goitre géant occupant toute la face antérieure surtout à gauche. La nasofibroscopie objective une mobilité normale des cordes vocales un larynx de morphologie normale et une trachée déviée à droite. Le scanner cervico-thoracique objective un goitre multihétéronodulaire avec des calcifications intranodulaires. Le gros nodule occupe tout le lobe thyroïdien gauche fait 9 cm, dévie la trachée à droite et plonge en endothoracique. Le bilan thyroïdien (TSH, Ft3 et FT4) est normal. Il n’ya pas d’adénopathies cervicales. La patiente a bénéficié d’une thyroïdectomie totale. Les suites opératoires sont simples et marqués par la reprise de l’alimentation normale et la disparition de la dyspnée. L’étude histologique est revenue en faveur d’une hypertrophie thyroïdienne bénigne. La patiente est mise sous L-Thyroxine et est adressée aux endocrinologues pour le suivi hormonal.

**Figure 1:**
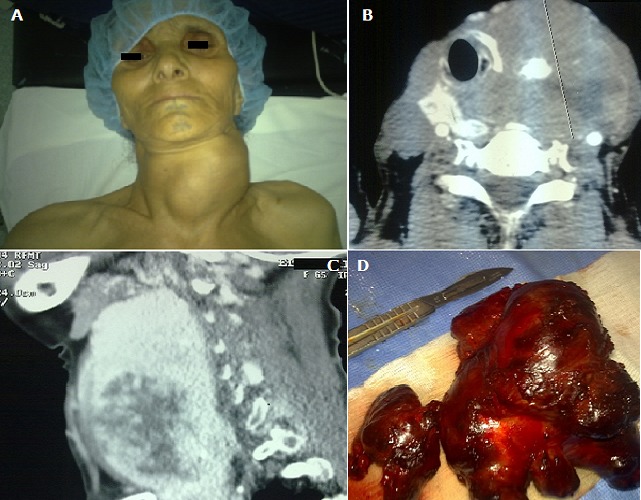
Goitre multinodulaire géant plongeant (A); TDM en coupe axiale objectivant un goitre contenant des macrocalcifications et refoulant la trachée à droite (B); TDM en coupe sagittale montrant un goitre plongeant en endothoracique (C); Pièce opératoire (D)

